# Autonomous Self-Adaptive and Self-Aware Optical Wireless Communication Systems

**DOI:** 10.3390/s23094331

**Published:** 2023-04-27

**Authors:** Maged Abdullah Esmail

**Affiliations:** Smart Systems Engineering Laboratory, Department of Communications and Networks Engineering, Prince Sultan University, Riyadh 11586, Saudi Arabia; mesmail@psu.edu.sa

**Keywords:** FSO, machine learning, turbulence, random forest, regressor, classifier, optical networks

## Abstract

The future age of optical networks demands autonomous functions to optimize available resources. With autonomy, the communication network should be able to learn and adapt to the dynamic environment. Among the different autonomous tasks, this work considers building self-adaptive and self-awareness-free space optic (FSO) networks by exploiting advances in artificial intelligence. In this regard, we study the use of machine learning (ML) techniques to build self-adaptive and self-awareness FSO systems capable of classifying the modulation format/baud rate and predicting the number of channel impairments. The study considers four modulation formats and four baud rates applicable in current commercial FSO systems. Moreover, two main channel impairments are considered. The results show that the proposed ML algorithm is capable of achieving 100% classification accuracy for the considered modulation formats/baud rates even under harsh channel conditions. Moreover, the prediction accuracy of the channel impairments ranges between 71% and 100% depending on the predicted parameter type and channel conditions.

## 1. Introduction

One variant of optical fiber communication is free space optics (FSO), which replaces the fiber medium of transmission with free space. FSO is advantageous over fiber as it reduces capital expenditures by eliminating the need for ground digging. Moreover, it is the only solution when fiber installation is impossible, such as on high-traffic roads and private properties. In addition, FSO provides high bandwidth, high security, and low power consumption, and is license-free [1,2].

The research community’s interest in improving the capacity and efficiency of optical communication networks has motivated them to consider using heterogeneous and dynamic networks, which enable the building of an autonomous network that is self-aware of the network status and self-adaptive [3,4].

Self-awareness is related to the availability and collection of knowledge about a system by that system. Such knowledge enables making intelligent decisions, which in turn leads to adaptive behavior, i.e., a self-adaptive system [5]. Therefore, the self-awareness function is considered a pre-requisite step for building a self-adaptive system. Understanding self-awareness and self-adaptive functions is the cornerstone of modeling, designing, and engineering future systems. The self-awareness concept has been applied in many disciplines, such as computing systems [6], artificial neural networks [5], Internet of Things networks [7], and image security [8].

In FSO systems, the link is time-varying due to outdoor environmental conditions such as fog, rain, and turbulence. Efficient performance monitoring techniques are required to propose suitable compensation techniques to mitigate such outdoor environmental conditions and build a self-awareness FSO network. Additionally, transmitting data adaptively to efficiently use channel capacity requires using adaptive modulation formats and baud rates, which necessitates self-adaptive functions in the network.

A self-adaptive FSO system can be achieved by employing functions such as the modulation format and baud rate classification. These functions enable the FSO system to explore available resources adaptively, maximizing channel capacity under the randomly time-variant outdoor channel. When the channel fluctuation is light, the system can transmit at a high modulation format and/or baud rate. However, if the channel becomes worse, the transmission rate reduces by using a lower modulation format and/or baud rate. To achieve this self-adaptive function, the FSO system should be intelligent and be able to determine the transmitted signal’s modulation format and baud rate to determine the appropriate action, such as choosing the modulation format/baud rate to demodulate the signal correctly.

On the other side, a self-awareness FSO system is important to determine the network status by calculating the amount of distortion in the received signal. Knowing the distortion amount enables the FSO system to select an appropriate mitigation technique. In addition, it helps in selecting the baud rate/modulation format type that fits the channel conditions.

Nowadays, machine learning (ML) is a hot topic in optical communication and almost every scientific field, with unlimited applications [7,9,10,11]. It replaces the complex mathematical modeling of the problem under consideration with intelligent algorithms that can extract the functional relationship with less complexity and provide high-accuracy decisions. Different ML techniques are proposed in the literature for modulation format classification, baud rate classification, and performance monitoring in radio frequency (RF) and optical communication networks [9,10,12]. The features extracted for training the model depend on the types of channel impairments.

Many ML techniques have been exploited in fiber-based optical communication networks that consider direct detection techniques instead of coherent detection, which is more complex and expensive. In [13], the principal component analysis (PCA) technique is used for the classification of three different modulation formats (return-to-zero on–off keying (RZ-OOK), non-RZ differential phase shift keying (NRZ-DPSK), and RZ-DPSK). The training features are extracted using the asynchronous single-channel sampling (ASCS) method. In [14], the authors applied a convolutional neural network (CNN) algorithm for the modulation classification of four modulation formats (RZ-OOK, NRZ-OOK, RZ-DPSK, and four-pulse amplitude modulation (4-PAM)). In this work, eye diagrams are exploited as features for training the ML model. The authors in [15] used asynchronous histogram (AH) as a feature for training a deep neural network (DNN) algorithm to classify three types of modulation formats (NRZ-OOK, 4-PAM, and 8-PAM). Note that the previous three proposed techniques were not used to classify the baud rate and were used to monitor a single impairment, which is OSNR. In [16], the authors used the CNN algorithm and synchronous delay-tap sampling (ADTS) as a feature extraction method to classify three different modulation formats (NRZ-OOK, RZ-OOK, and NRZ-DPSK). In addition, the technique is used for baud rate classification and monitoring three fiber-based impairments, which are the OSNR parameter, chromatic dispersion (CD) parameter, and differential group delay (DGD) parameter. The same impairments were monitored in [17] with baud rate classification but the classification was achieved for three other modulation formats (RZ-OOK, DP-RZ-quadrature PSK (QPSK), and DP-NRZ-16 quadrature amplitude modulation (QAM)). In addition to these techniques, there are other classification/monitoring techniques that exploit coherent optical receivers instead of direct detection receivers [10].

The application of ML for free-space-based optical networks is still in its infancy. In FSO, space-based impairments differ from those in fiber, requiring investigation of the suitability of available ML techniques for FSO systems. A few ML techniques have been proposed in the literature for FSO systems. CNN ML is used to monitor turbulence impairment in the orbital angular modes (OAMs) transmitted over an FSO channel in [18,19]. Visibility range prediction investigation is reported in [20,21] for dusty and foggy channel conditions, respectively, using CNN and support vector machine (SVM) ML techniques. In [22], SVM and CNN ML models were used to monitor three channel parameters (turbulence, OSNR, pointing errors), with training features extracted using AAH and ADTS methods. The FSO system exploited on–off keying (OOK) as a modulation format with a 10 Gbps transmission speed. Note that all reported techniques performed only performance monitoring without the modulation format/baud rate classification. Table 1 compares the current work with the literature studies.

In this work, we propose using ML for building autonomous FSO systems with self-awareness and self-adaptiveness functions. The exploited ML model will be used for the (1) modulation format classification, (2) baud rate classification, (3) turbulence impairment parameter monitoring, and (4) OSNR parameter monitoring. To the best of our knowledge, this is the first time a work utilizes ML to perform modulation classification and the four different tasks in FSO systems. In a previous work [20,21], only NRZ OOK modulation is considered for channel impairments monitoring. In this work, four modulation formats are considered, i.e., NRZ OOK, RZ OOK, 4-PAM, and 8-PAM. Moreover, four baud rates are considered, i.e., 10 Gbaud, 20 Gbaud, 30 Gbaud, and 40 Gbaud. Such baud rate values and modulation format types are suitable for intensity modulation/direct detection (IM/DD) FSO systems, which are currently the practical versions of FSO.

The remainder of the paper is organized as follows. In Section 2, we discuss the FSO communication system and the channel model. Section 3 describes the simulation setup, settings of the used ML technique, and data generation and processing. Section 4 presents the result; finally, we conclude in Section 5.

## 2. FSO System and Channel Model

In traditional FSO systems, IM/DD techniques are used instead of coherent techniques. This is because coherent techniques are practically hard to implement due to the need for precise alignment of the FSO link so that the light at the receiver side can be coupled into the fiber with low-power loss. Keeping the FSO link alignment of a coherent FSO system is difficult in a time-varying channel, such as that of terrestrial FSO, making the implementation impractical. Therefore, traditional intensity modulation and direct detection (IM/DD) links are widely used.

The traditional IM/DD FSO system is modeled mathematically by [23]

(1) $$y=Rh_{l}x+n,$$

where *R* is the photodetector responsivity parameter, $h_{l}$ is the channel state arising from atmospheric turbulence, *x* is the transmitted signal intensity, and *n* is an additive white Gaussian noise. The noise originates from using a boost amplifier on the transmitter side to compensate for link power losses due to beam spreading and signal scattering. The atmospheric turbulence causes signal fluctuation, known as scintillation, which results from the air’s refractive index fluctuation along the propagation path.

The signal intensity in a weak to strong turbulent medium is modeled by the gamma–gamma distribution, which is defined mathematically by [24]

(2) $$f\left(I\right)=\frac{2{\left(\alpha \beta \right)}^{(\alpha +\beta )/2}}{\Gamma (\alpha )\Gamma (\beta )}I^{(\alpha +\beta )/2-1}K_{\alpha -\beta }\left(2\sqrt{\alpha \beta I}\right),$$

where Γ(.) and $K_{\alpha -\beta }$ are the Gamma function and the modified Bessel function of the second kind with order α−β, respectively. The parameters α and β have an impact on the shape of the distribution function. Their values are given by the expression [24]

(3) $$\delta ={\left[\exp\left(\frac{a\sigma_{1}^{2}}{{\left(1+b\sigma_{1}^{12/5}\right)}^{c}}\right)-1\right]}^{-1},$$

where α=δ for a=0.49, b=1.11, and c=7/6, and β=δ for a=0.51, b=0.69, and c=5/6. The parameter $\sigma_{1}^{2}$ is the Rytov variance. The severity of turbulence is defined by the index of the refraction structure parameter, $C_{n}^{2}$, which is given by [24]

(4) $$C_{n}^{2}=\frac{\sigma_{1}^{2}}{1.23k^{7/6}L^{11/6}},$$

where *k* is the optical wave number and *L* is the link length. The turbulence parameter $C_{n}^{2}$ has values ranging from $10^{-17}$ m${}^{-2/3}$ for weak turbulence to $10^{-13}$ m${}^{-2/3}$ for strong turbulence. The turbulence and the system noise will be considered the source of the signal distortion. ML techniques will be exploited to predict the turbulence and noise parameters. We assume that the FSO link is aligned perfectly so the pointing errors are neglected.

## 3. Simulation Setup Description

### 3.1. Simulation Setup

**Figure 1:**
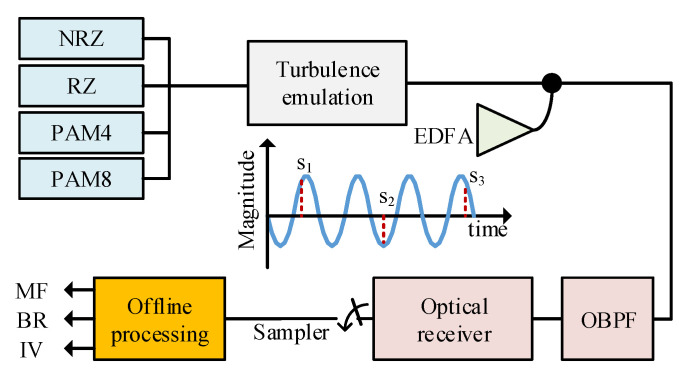
Simulation setup of the FSO communication system. OBPF: optical bandpass filter, EDFA: erbium-doped fiber amplifier, MF: modulation format, BR: baud rate, IV: impairment value.

Because the FSO channel status is random and unpredictable, obtaining enough signal measurements under different turbulence statuses in a reasonable time is difficult. Additionally, it is challenging to repeat measurements under the same channel conditions. Therefore, much research in the literature uses powerful simulators to emulate outdoor channel conditions. In this work, the Transmission Maker 11.1 simulator is used to build the training dataset of the ML model. This simulator is a powerful tool for designing and analyzing optical communication systems. Four different optical transmitters are used to generate an optical signal at four different modulation formats (NRZ, RZ, 4-PAM, and 8-PAM) and at different baud rates. The optical signal is generated using a laser diode (LD) at a 1550 nm wavelength and amplified using an optical amplifier to compensate for the power loss over the channel. The output of the optical transmitter is sent over the FSO turbulence emulator. Then the output signal is corrupted with ASE noise using an erbium-doped fiber amplifier (EDFA). Before detection, the signal is filtered using an optical bandpass filter (OBPF). The signal is then detected using an optical receiver that uses a PIN photodiode, which converts the optical signal into an electrical signal. The electrical output signal is filtered and then sampled before processing it offline. The offline process includes an ML model that can provide information about the classes of the modulation format and baud rate and the amount of impairment in the optical signal. The simulation setup is illustrated in Figure 1.

**Figure 2:**
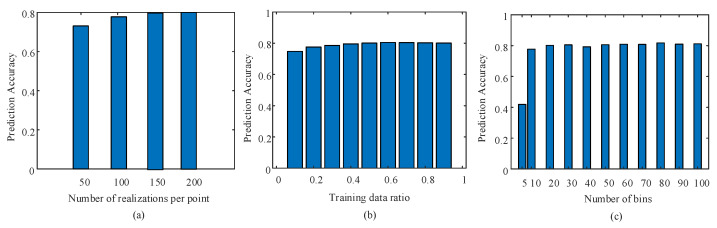
Prediction accuracy of the $C_{n}^{2}$ parameter versus (**a**) the number of realizations per point, (**b**) the training data ratio, and (**c**) the number of AAH bins. The signal is 8-PAM with a 10 Gbaud transmission speed and OSNR = 10 dB.

To create a powerful ML model, the training data should include weak to strong channel conditions. The ASE impairment is defined by the OSNR parameter from 10 dB to 18 dB with 2 dB steps (5 points). The turbulence impairment is defined by the $C_{n}^{2}$ parameter with the values $10^{-17}$ m${}^{-2/3}$, $10^{-16}$ m${}^{-2/3}$, $10^{-15}$ m${}^{-2/3}$, $10^{-14}$ m${}^{-2/3}$, and $10^{-13}$ m${}^{-2/3}$ (5 points) covering weak to strong turbulence. For each parameter value, several realizations are generated to train and test the ML model. To determine the appropriate number of realizations per parameter to use for training and testing the ML model, we trained and tested the proposed model at different numbers of realizations, as shown in Figure 2a. The results show that the best number of realizations per parameter is 150. This provides the highest prediction/classification accuracy. Hence, a dataset of 1500 realizations (150 realizations per point × 5 points per parameter × 2 parameters) is generated for training and testing the proposed model. This dataset is divided into two parts; one for training and one for testing. To determine the percentage of the dataset for training, we investigated in Figure 2b the performance accuracy for predicting the turbulence parameter of an 8-PAM signal with OSNR = 10 dB and 10 Gbaud speed. The results show that the best selection of the training percentage is 60%, which results in the highest achievable accuracy. Therefore, out of the 150 realizations per parameter, 90 realizations are used for training and the remaining 60 for testing.

### 3.2. Features Extraction

In traditional IM/DD FSO systems, only amplitude modulation formats can be used since DD does not preserve phase information. Therefore, in this work, we considered four modulation formats that include amplitude information. Since the information in the optical signal is modulated in the amplitude, it is obvious that the best feature for training an ML model is the one that preserves the amplitude information. In this work, the amplitude histogram is exploited as a feature for training the proposed model. Instead of sampling the signal at a high speed, which increases the acquisition system cost, an asynchronous amplitude histogram (AAH) is considered. Each realization is sampled at 500 Msample/s, which is a low sampling speed that helps in building a cost-effective ML model. The signal is sampled asynchronously as illustrated in Figure 1 by the samples $s_{1},s_{2}$, and $s_{3}$. Each realization is represented by 8192 samples. In addition to the low cost of the acquisition system when using the AAH feature, AAH also has the advantage of not requiring timing recovery at the receiver, eliminating the need to add additional hardware [10].

**Figure 3:**
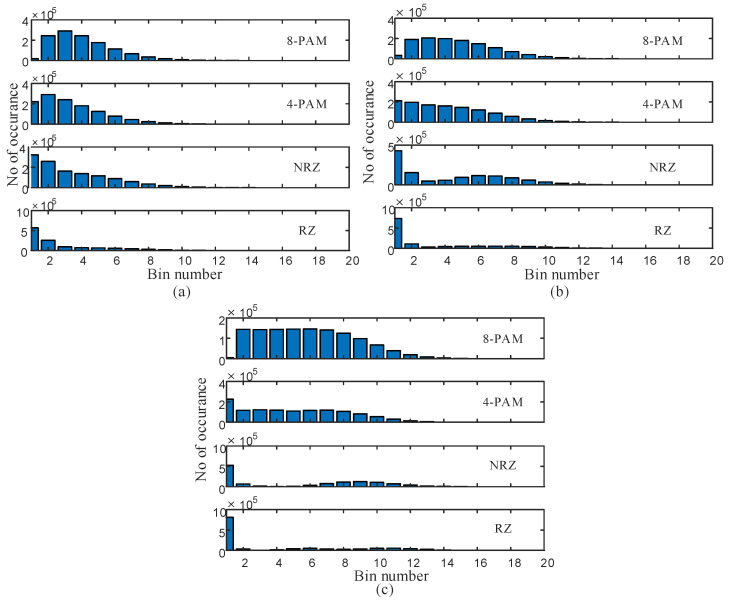
AAH features with 20 bins for 4 different modulation format signals under different turbulence conditions: (**a**) $C_{n}^{2}=10^{-17}$ m${}^{-2/3}$, (**b**) $C_{n}^{2}=10^{-15}$ m${}^{-2/3}$, and (**c**) $C_{n}^{2}=10^{-13}$ m${}^{-2/3}$.

Figure 3 shows the AAH features for the four modulation format signals under different values of channel turbulence that cover weak to strong turbulence. It is clear that the AAH features are somewhat different for each modulation format (under the same impairment or different values for turbulence). For example, in Figure 3a, the 4-PAM signal has a different AAH feature than the three other modulation formats. In addition, the AAH feature of the 4-PAM signal is different under the three different turbulence conditions, as shown in Figure 3a–c. Therefore, the AAH feature is a powerful tool in this work that will simplify building the autonomous FSO system. The amount of correlation between the different AAH features will determine the accuracy of the ML model.

The number of bins that are used in AAH determines the accuracy of prediction/ classification. Figure 2c shows a plot of the prediction accuracy versus the number of bins used in AAH for the 8-PAM signal at a 10 Gbaud transmission speed. For a very low number of bins (less than 10), the accuracy is highly degraded. However, for a number of bins equal to or higher than 10, the accuracy is highly improved. For 20 bins, the accuracy is 80.2%, which is 1% less than the best accuracy at 100 bins. The choice of 20 bins reduces system complexity as it requires lower data processing.

### 3.3. Machine Learning Algorithm

**Figure 4:**
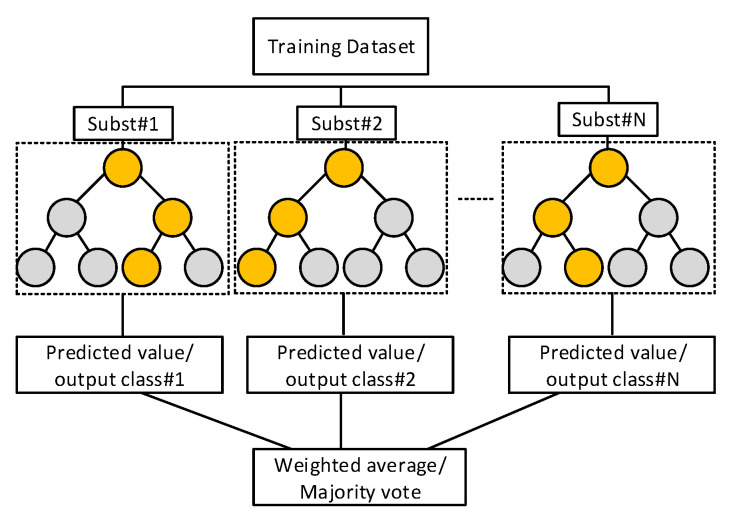
Random forest ensemble conceptual framework.

In this work, we consider the random forest (RF) ensemble algorithm for both modulation format/baud rate classification and impairment regression. Its principle is based on building a number of independent decision trees (DTs) during the training phase; each one is trained individually by selecting a random subset from the whole training dataset. This randomness ensures having DTs that are independently trained [10,25]. The randomness training ensures that if some DTs would produce less accurate results, many could produce more accurate results. Hence, instead of using a single DT, ensemble learning that uses a group of DTs ensures stable results with better classification/prediction accuracy [26,27]. After training, the predictions from all individual DTs will be averaged in the case of prediction RF. For classification, a majority vote is taken, where the class with the maximum number of votes is selected. Figure 4 illustrates the conceptual framework of the RF ensemble algorithm that includes *N* DTs.

## 4. Results and Discussions

### 4.1. Modulation Format Classification

**Figure 5:**
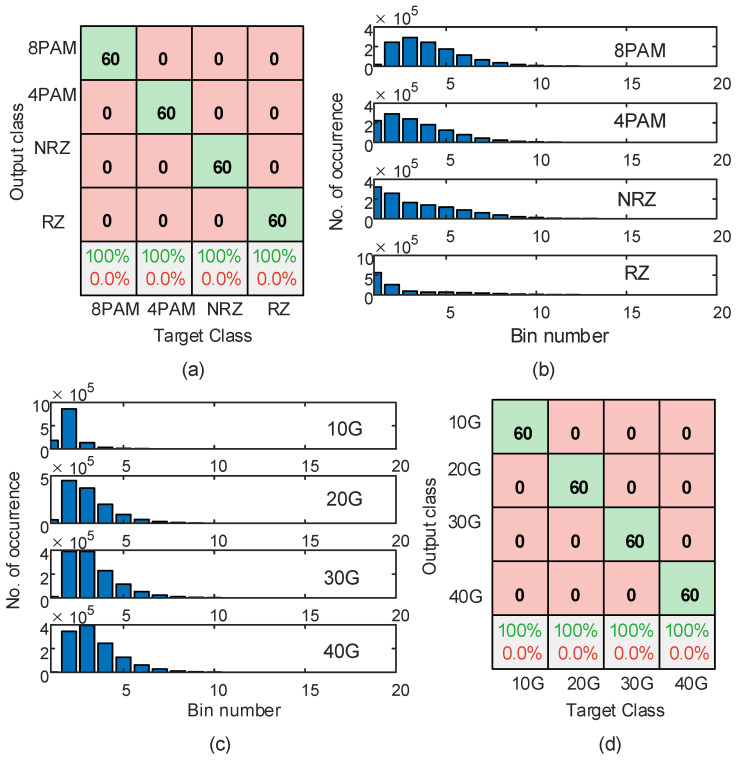
(**a**) Confusion matrix for classifying the modulation format type, (**b**) AAH features for different modulation formats, (**c**) AAH features for the 8-PAM signal with different baud rates, and (**d**) confusion matrix for classifying the baud rate. Each signal is corrupted with OSNR = 10 dB and $C_{n}^{2}=10^{-13}$ m${}^{-2/3}$.

The RF modulation format classifier is used to classify the four modulation formats, which are NRZ, RZ, 4-PAM, and 8-PAM, under different system conditions. The confusion matrix that is shown in Figure 5a is used to illustrate the accuracy of the modulation format classification by comparing the target class with the model’s output class. The diagonal cells show the number of correctly identified modulation formats among the total number in the test data, i.e., among the 60 realizations used for model testing for each parameter. The off-diagonal cells show the number of misclassified modulation formats. The last row shows the accuracy percentage of predicting the modulation format (top green) and the percentage of misclassifying it (bottom red).

In Figure 5a, we consider a harsh channel defined by 10 dB OSNR and $C_{n}^{2}=10^{-13}$ m${}^{-2/3}$. The confusion matrix indicates that the accuracy of classification is 100% for each class, i.e., the modulation format. This high accuracy is due to each class’s distinct AAH features, as illustrated in Figure 5b. When the OSNR parameter and/or the turbulence parameter improved, i.e., the channel condition improves, the classification accuracy does not degrade. Such results usually appear when using powerful training features in classification problems [10].

### 4.2. Baud Rate Classification

In adaptive networks, the signal’s baud rate changes according to the severity of the channel. In this subsection, we consider four different baud rates: 10, 20, 30, and 40 Gbaud. The RF classifier is used to identify the baud rate in the received signal after being distorted by the channel. Figure 5c shows the AAH features for an 8-PAM signal with different baud rates under harsh channel conditions defined by 10 dB OSNR and $C_{n}^{2}=10^{-13}$ m${}^{-2/3}$. The AAH features in Figure 5c show that the four baud rates have different AAH features, simplifying the classifier function to identify them. The results in Figure 5d show that the classifier can classify each baud rate with 100% accuracy. Similar results, i.e., 100% accuracy, are achieved with the other signal modulation formats (4-PAM, NRZ, and RZ) under lighter weather conditions.

### 4.3. Impairments Regression

**Figure 6:**
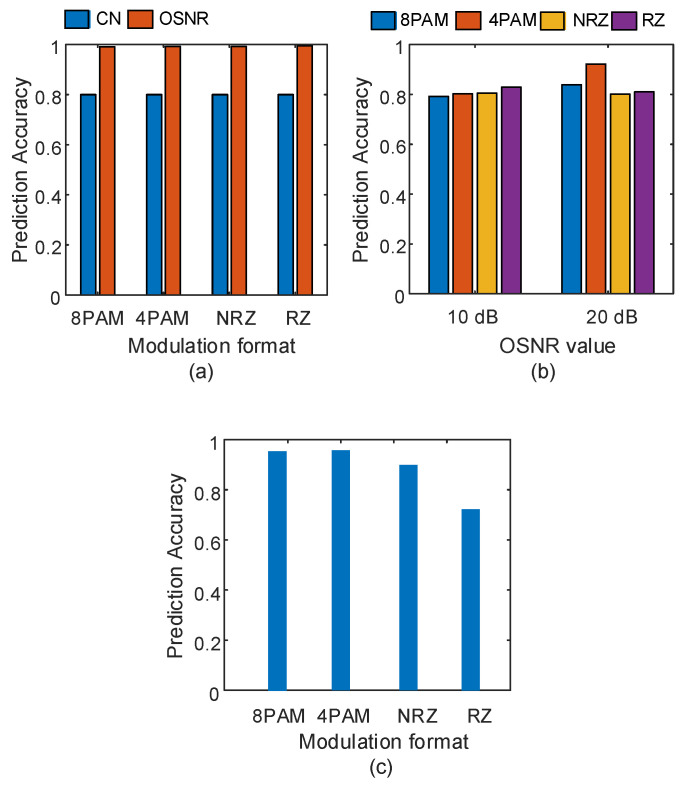
(**a**) Prediction accuracy of $C_{n}^{2}$ and OSNR parameters, (**b**) $C_{n}^{2}$ parameter prediction at two different values of the OSNR parameter, (**c**) OSNR parameter prediction at $C_{n}^{2}=10^{-13}$ m${}^{-2/3}$. The signals are transmitted at 10 Gbaud.

In this subsection, we discuss the performance of the ML model in predicting the amount of impairment in the optical signal. For this purpose, the RF ML algorithm is used as a regressor rather than a classifier. To show the regression accuracy, we exploit the coefficient of determination metric, which is defined by [28]

(5) $$\rho =1-\frac{\sum_{n=1}^{N}{\left(m_{n}-{\hat{m}}_{n}\right)}^{2}}{\sum_{n=1}^{N}{\left(m_{n}-\bar{m}\right)}^{2}},$$

where $m_{n}$ and ${\hat{m}}_{n}$ are the actual and estimated data, $\bar{m}$ is the sample mean, and *N* is the total number of test samples. This metric takes values between 0 and 1. The prediction accuracy improves when ρ→1. Moreover, the prediction accuracy deteriorates when ρ→0. Therefore, the model’s output exactly matches the target value when ρ=1. Moreover, the model cannot predict the true target value when ρ=0. First, we consider predicting the two impairment parameters, i.e., the OSNR parameter and turbulence parameter, individually. Figure 6a shows the prediction accuracy for the OSNR parameter and the turbulence parameter in optical signals with different types of modulation formats, each one transmitted at 10 Gbaud. For the OSNR parameter, the regressor is able to predict its value with 100% prediction accuracy. This prediction accuracy drops to 80% for the turbulence parameter prediction regardless of the type of modulation format. This is because, under light turbulence conditions, the similarity (i.e., correlation, between the AAH features) increases, which complicates the regressor prediction’s capability.

Next, we study the case of predicting the turbulence parameter when the signal is also corrupted by certain values of ASE noise. Two different values are considered for the OSNR parameter: 10 and 20 dB. The results in Figure 6b show that the prediction accuracy improves with 8-PAM and 4-PAM modulation formats when the OSNR value improves from 10 to 20 dB. For NRZ and RZ modulation format signals, the prediction accuracy almost shows no improvement. The minimum prediction accuracy is 79%, and the best-achieved accuracy is 92%. In Figure 6c, we illustrate the prediction accuracy of the OSNR parameter when the signal is corrupted by severe turbulence. The prediction accuracy of the OSNR parameter drops from 95% for 8-PAM and 4-PAM signals to 90% and 71% for NRZ and RZ signals, respectively. This is due to the increase in similarity between the AAH features for NRZ and RZ signals.

### 4.4. Performance Comparison

In this section, we first compare the performance of the proposed model with the DT model, which is an ML-based algorithm. Then, we compare the proposed model with a non-ML model.

**Figure 7:**
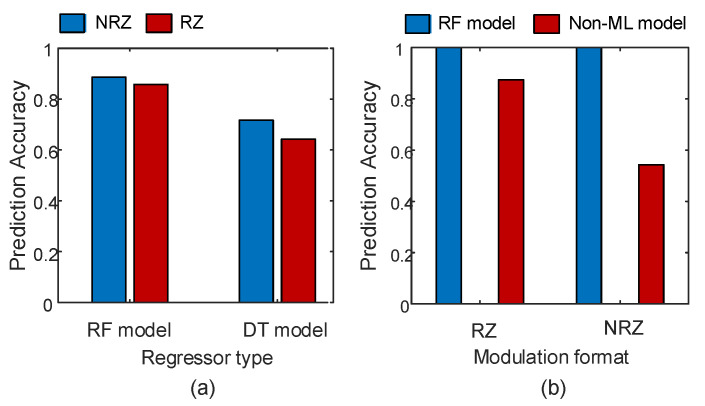
(**a**) Comparison between RF and DT ML models for OSNR parameter prediction at $C_{n}^{2}=10^{-13}$ m${}^{-2/3}$ and two different modulation formats, and (**b**) a comparison of the RF ML model with a non-ML model using two different modulation formats. The signals are transmitted at 10 Gbaud.

Figure 7a compares the performance between the RF ensemble algorithm and DT algorithm for predicting the OSNR parameter when the signal is also corrupted by a harsh turbulence environment, defined by models for OSNR parameter prediction at $C_{n}^{2}=10^{-13}$ m${}^{-2/3}$. The prediction results show that the prediction accuracy of RF is higher than DT by 8% and 21% when the signal uses NRZ and RZ modulation formats, respectively. These results illustrate the power of using a forest of DTs instead of using a single DT.

Next, we compare the RF ML model with a non-ML classical model for OSNR parameter prediction. There are numerous classical methods in the literature for monitoring OSNR parameters [10]. One of them is based on calculating the Q-factor parameter. The Q-factor parameter is calculated using [29]

(6) $$Q=\frac{|\mu_{1}-\mu_{0}|}{\sigma_{1}+\sigma_{0}},$$

where $\mu_{1}$ and $\sigma_{1}$ are the mean and standard deviations of the “1”, respectively, of the amplitude histogram, while $\mu_{0}$ and $\sigma_{0}$ are the mean and standard deviations of the “0”, respectively, of the amplitude histogram. To find the prediction accuracy of the OSNR parameter, we first define the OSNR parameter in terms of the Q-factor parameter using [30]

(7) $$Q\left(dB\right)=OSNR+10log\left(B_{o}/B_{c}\right),$$

where $B_{o}$ and $B_{c}$ are the optical bandwidth of the photodiode and the electrical bandwidth of the receiver filter, respectively. Figure 7b shows the OSNR parameter prediction accuracy results of the RF ML proposed model and the non-ML model. For the RZ modulation format, the proposed model achieved 100% prediction accuracy while the non-ML model achieved 87% prediction accuracy. For the NRZ modulation format, the proposed model maintained the same prediction accuracy. However, the non-ML model’s prediction accuracy dropped to 54%. This reflects the power of using ML compared to non-ML models.

## 5. Conclusions

In this work, we propose using ML techniques for building self-aware and self-adaptive autonomous optical wireless communication systems. The RF ML technique is exploited for both the modulation format/baud rate classification and channel impairment prediction. Simulation results showed that the proposed model can classify the modulation format/baud rate with 100% accuracy. However, the prediction accuracy results show that the model can achieve accuracy between 71% and 100%, depending on the modulation format type, channel conditions, and the type of the predicted parameter. To understand the power of using ML models, we compared the proposed ML technique with a non-ML technique. The results show that the proposed ML technique outperforms the non-ML technique. Therefore, ML techniques are expected to be a building block in future autonomous optical networks.

## Figures and Tables

**Table 1:** Literature review of modulation format/baud rate classification and channel parameter monitoring techniques using the direct detection technique.

Refs.	Channel Type	ML Model	Training Feature	Modulation Classification?	Baud Rate Classification?	Monitored Parameters
[13]	Fiber	PCA	ASCS	RZ-OOK NRZ-DPSK RZ-DPSK	No	OSNR
[14]	Fiber	CNN	Eye diagram	RZ-OOK NRZ-OOK RZ-DPSK 4-PAM	No	OSNR
[15]	Fiber	DNN	AH	NRZ-OOK 4-PAM 8-PAM	No	OSNR
[16]	Fiber	CNN	ASTS	NRZ-OOK RZ-OOK NRZ-DPSK	Yes	OSNR CD DGD
[17]	Fiber	PCA	ADTS	RZ-OOK DP-RZ-QPSK DP-NRZ- 16QAM	Yes	OSNR CD DGD
[18]	FSO	CNN	OAM modes	No	No	Turbulence
[19]	FSO	CNN	OAM modes	No	No	Turbulence
[20]	FSO	CNN	OAM modes	No	No	Visibility
[21]	FSO	SVM	OAM modes	No	No	Visibility
[22]	FSO	SVM+ CNN	AAH+ ADTS	No	No	Turbulence, OSNR, Pointing errors
This work	FSO	RF	AAH	NRZ OOK RZ OOK 4-PAM 8-PAM	Yes	Turbulence OSNR

## Data Availability

Not applicable.
